# Methotrexate impaired in-vivo matured mouse oocyte quality and the possible mechanisms

**DOI:** 10.1186/s12860-020-00298-7

**Published:** 2020-07-03

**Authors:** Ning Tian, Dan-yu Lv, Ji Yu, Wan-yun Ma

**Affiliations:** 1 Physical Science and Technical College, Shenyang Normal University, No. 253 Huanghe North Street, Huanggu District, Shenyang City, 110034 Liaoning Province China; 2 Department of Histology and Embryology, School of Basic Medical Sciences, Peking University Health Science Center, No. 38 Xueyuan Road, Haidian District, Beijing, 100191 China; 3 State Key Laboratory of Low-Dimensional Quantum Physics, Department of Physics, Tsinghua University, Haidian District, Beijing, 100084 China

**Keywords:** Methotrexate, Oocyte quality, Folate metabolism, Chromosome, Methylation modification

## Abstract

**Background:**

Methotrexate (MTX) is an antifolate agent which is widely used in clinic for treating malignancies, rheumatoid arthritis and ectopic pregnancy. As reported, MTX has side effects on gastrointestinal system, nervous system and reproductive system, while its potential damages on oocyte quality are still unclear. It is known that oocyte quality is essential for healthy conception and the forthcoming embryo development. Thus, this work studied the effects of MTX on the oocyte quality.

**Results:**

We established MTX model mice by single treatment with 5 mg/Kg MTX. Both morphological and molecular biology studies were performed to assess the in-vivo matured oocytes quality and to analyze the related mechanisms. The in-vivo matured oocytes from MTX-treated mice had poor in-vitro fertilization ability, and the resulting embryo formation rates and blastocyst quality were lower than the control group. We found that the in-vivo matured MTX-treated mouse oocytes displayed abnormal transcript expressions for genes of key enzymes in the folate cycles. MTX increased the rate of abnormal chromosome alignment and affected the regulation of chromosome separation via disrupting the spindle morphology and reducing the mRNA expressions of MAD2 and Sgo1. MTX reduced the DNA methylation levels in the in-vivo matured oocytes, and further studies showed that MTX altered the expressions of DNMT1 and DNMT 3b, and may also affect the levels of the methyl donor and its metabolite.

**Conclusions:**

MTX impaired the in-vivo matured mouse oocyte quality by disturbing folate metabolism and affecting chromosome stability and methylation modification.

## Background

Methotrexate (MTX) is a folate antagonist which is transferred into the cell by the solute carrier family 19 (SLC19A) and competitively inhibits the dihydrofolate reductase (DHFR) activity [1–4]. Thus, MTX reduces catalytic conversion of dihydrofolate (DHF) to tetrahydrofolate (THF), namely seriously disturbing the folate metabolism [1, 2, 5]. The several important enzymes in the folate pathway (including serine hydroxymethyl transferase (SHMT), 5,10-methylene THF reductase (MTHFR), methionine synthase reductase (MTRR), methionine adenosyl transferase (MAT), cystathionine β-synthase (CSB), and so on [6]; as shown in Fig. S1) may also be indirectly inhibited by MTX. Additionally, the folate metabolites (such as purine, thymidine, S-adenosylmethionine (SAM); as shown in Fig. S1), which are essential for the DNA and RNA synthesis and methylation modification [7, 8], may also be affected by the MTX toxicity.

Due to the biological activities of MTX, MTX is widely used in clinic for the treatment of various neoplastic diseases (such as leukemia and lymphoma), various autoimmune and inflammatory disorders (rheumatoid arthritis and psoriasis, for example) [9–12]. For the female patients, MTX also is the common medicament used for the termination of pregnancy, ectopic pregnancy, and other uterine diseases [13, 14]. It is not desirable that MTX causes damages to various organs including stomach, intestine, hematologic system, liver, lung and central nervous system [15–19]. In addition, MTX also has side effects on reproductive system usually with defective oogenesis and spermatogenesis which may result in infertility [20, 21]. Until now, the studies of the association between female fertility and MTX toxicity have been mainly focused on analyzing the effects of MTX on follicle stimulating hormone, antral follicle count, oocyte yield, ovarian reserve, ovarian responsiveness, and so on [22–24]. It is known that oocyte quality is critical for female fertilization and embryonic development. However, the association between MTX toxicity and oocyte quality is still unclear.

The purpose of this study was to explore the effects of MTX on the oocyte quality and its possible mechanisms. We firstly confirmed the effects of MTX on in-vivo matured oocyte quality by analyzing the in-vitro fertilization ability and the resulting embryo formation. Considering the metabolic pathway of MTX, the transcript expressions for genes of the key enzymes in the folate cycles were examined. Chromosome stability is a major factor in developmental potential of oocytes. Therefore, the chromosome alignment and the separation regulation were analyzed in this work. It is known that the correct separation of chromosomes is regulated by spindle formation, spindle assembly checkpoint (SAC) and cohesin dissociation [25, 26]. Mitotic arrest deficient 2 (MAD2) is the main component of SAC, and Shugoshin 1 (Sgo1) plays a key role in protecting cohesion [25–27]. Thus, this work examined the spindle morphology and the mRNA levels of MAD2 and Sgo1 to determine the effects of MTX on chromosome separation. DNA methylation is an important epigenetic modification, which has close link with gene expression and chromatin organization [28, 29]. The folate metabolite, SAM, is required as methyl donor for methyl metabolism [7]. DNA methyltransferases (DNMTs) have the functions of methylating DNA, namely, add a methyl from SAM to the carbon 5 position of CpG dinucleotide [30]. Thus, to determine the MTX-induced changes on DNA methylation modification, we also examined the expressions of DNMTs and the concentrations of SAM and its metabolite, S-adenosylhomocysteine (SAH). Our observations exhibit insights into the origin of MTX toxicity on oocyte quality and developmental competence.

## Results

### MTX reduced the in-vitro fertilization ability of in-vivo matured oocytes and the embryo development potential

As seen in Table 1, the in-vitro fertilization rate of in-vivo matured oocytes from MTX model mice was 59.9% ± 3.7%, which was much lower than 81.5% ± 2.1% in the control group (*p* < 0.05). The resulting 2-cell rate, 8-cell rate and blastocyst rate in the MTX group were also greatly reduced (46.9% ± 2.2, 29.9% ± 2.1 and 21.2% ± 3.3% in the MTX group versus 76.7% ± 1.8, 59.4% ± 2.5 and 48.8% ± 3.1% in the control group). Blastocyst quality was assessed by the ratio of ICM cells to total cells. In the control group, the ICM cell number and the total cell number were 14.6 ± 1.7 and 46.1 ± 2.2, the radio of which was 31.7% ± 4.0%. The corresponding estimates in the MTX group were 11.5 ± 1.9, 45.5 ± 2.0 and 25.8% ± 3.9%. There were significant differences in the ICM cell number and the radio between the control and MTX groups.

**Table 1:** Effects of MTX on the oocyte fertilization and the fertilized embryo formation

	Fertilization rate (%)	2-cell rate (%)	8-cell rate (%)	Blastocyst rate (%)	No. of ICM; No. of total cells; ratio (%)
Control group (total number of oocytes, No.168)	81.5 ± 2.1	76.7 ± 1.8	59.4 ± 2.5	48.8 ± 3.1	14.6 ± 1. 7 46.1 ± 2.2 31.7 ± 4.0
MTX group (No.124)	59.9 ± 3.7*	46.9 ± 2.2*	29.9 ± 2.1*	21.2 ± 3.3*	11.5 ± 1.9* 45.5 ± 2.0 25.8 ± 3.9*

* *p* < 0.05, There is significant difference between the MTX and control groups

### MTX reduced the mRNA transcriptional expressions for the key enzymes involved in the folate cycles

**Fig. 1:**
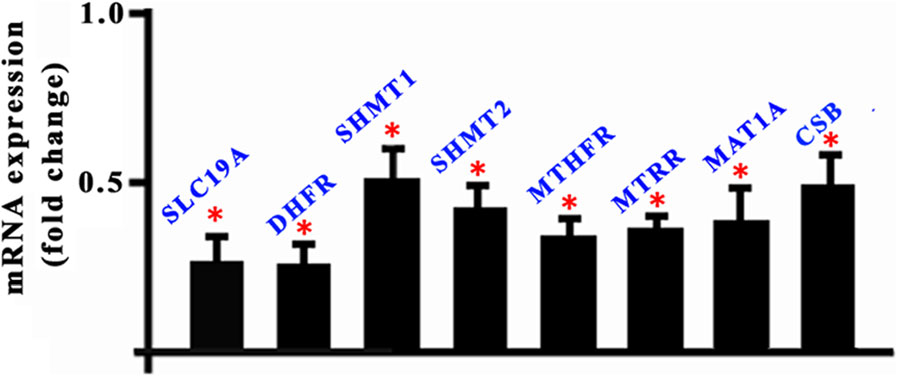
Transcript expressions for the key enzymes of the folate metabolism in the MTX-treated mouse oocytes. * *p*<0.05

There were no significant differences on the total RNA quantity between control (about 0.39 ± 0.03 ng RNA per oocyte) and MTX (about 0.38 ± 0.02 ng RNA per oocyte) groups. Real-time PCR results showed that the mRNA expressions of the key enzymes in the folate cycles, namely, SLC19A, DHFR, SHMT1, SHMT2, MTHFR, MTRR, MAT1A and CSB were significantly reduced in the MTX-treated mouse matured oocytes (0.259 ± 0.086, 0.25 ± 0.069, 0.509 ± 0.095, 0.491 ± 0.069, 0.397 ± 0.061, 0.405 ± 0.043, 0.491 ± 0.112, 0.586 ± 0.095 versus 1.0; Fig. 1).

### MTX increased the errors in the chromosome alignment

**Fig. 2:**
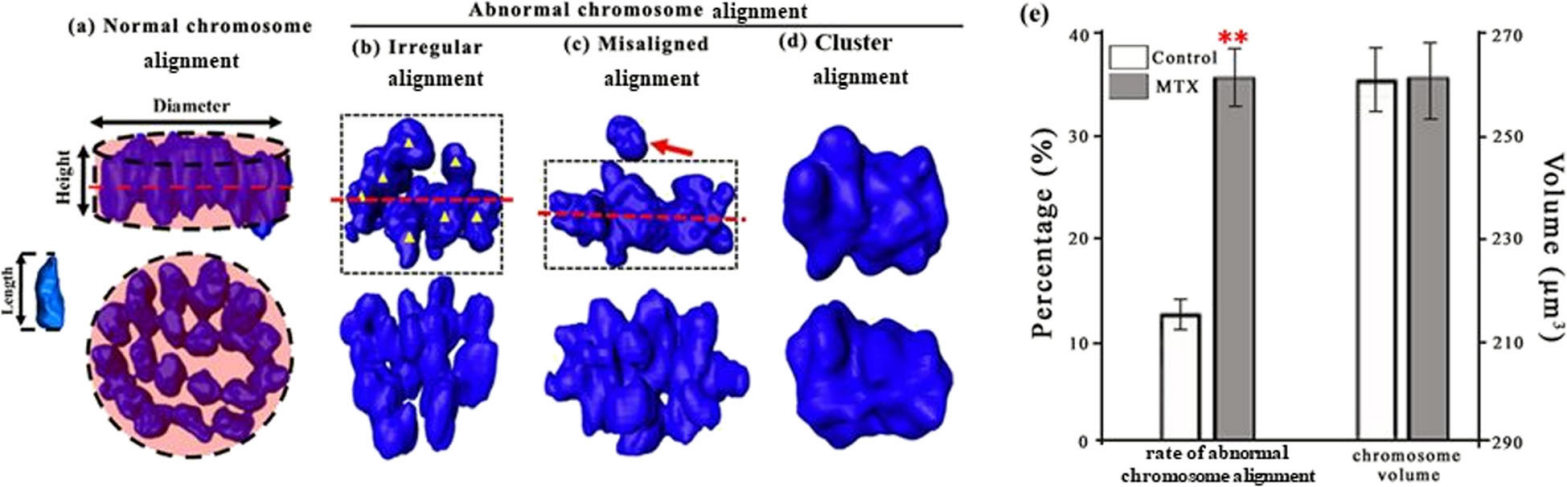
Effects of MTX on the chromosome alignment. Chromosomes were visualized using Hoechst 33342, and were reconstructed using Amira 5.2. Chromosome alignments were observed from the directions parallel and perpendicular to the equatorial plate. The dot line indicates the equatorial plate. **a** Normal chromosome alignment in the in-vivo matured oocyte is rather regular and looks like a disc-shaped plate, namely, all the chromosomes are neatly arranged at the equatorial plate. We used a short circular cylinder as the model of chromosome plate, and the volume and surface area of this cylinder were same to the chromosome pate. The cylinder height (H) and univalent length (L) were measured. The normal alignment had the characteristic of H ≤ 2 L. **b** The irregular alignment had the characteristic of H>2 L. **c** The misaligned alignment was that individual univalents were not on the equatorial plate, while others were still along the plate. The red arrow indicates the misaligned chromosome. **d** The chromosome alignment of all the chromosomes clustered together in a great mass was defined as cluster alignment, and the equatorial plate can’t be found. The rates of abnormal chromosome alignment and chromosome volumes were shown in **e**. Chromosome volume was estimated by the spatial area occupied by fluorescence, and was calculated using Amira 5.2. The oocyte numbers for analyzing the rates of abnormal chromosome alignment were 384 in the control group, and 275 oocytes in the MTX group. ** *p*<0.01

In this work, chromosome alignments at the second metaphase of mouse oocytes (Fig. 2a-d) were divided into normal alignment and abnormal alignment (including irregular alignment, misaligned alignment and cluster alignment). The rate of abnormal alignment in the MTX group was significantly higher than that in the control group (35.0% ± 2.1% versus 12.8% ± 1.2%; Fig. 2e). The oocyte sizes in both MTX and control groups were similar (74.1 ± 5.5 μm versus 75.1 ± 5.0 μm), also, the spatial area occupied by the chromosome fluorescence did not significantly differ (263.2 ± 6.5 μm^3^ versus 263.7 ± 8.2 μm^3^; Fig. 2e).

### MTX affected the regulation of chromosomes separation

**Fig. 3:**
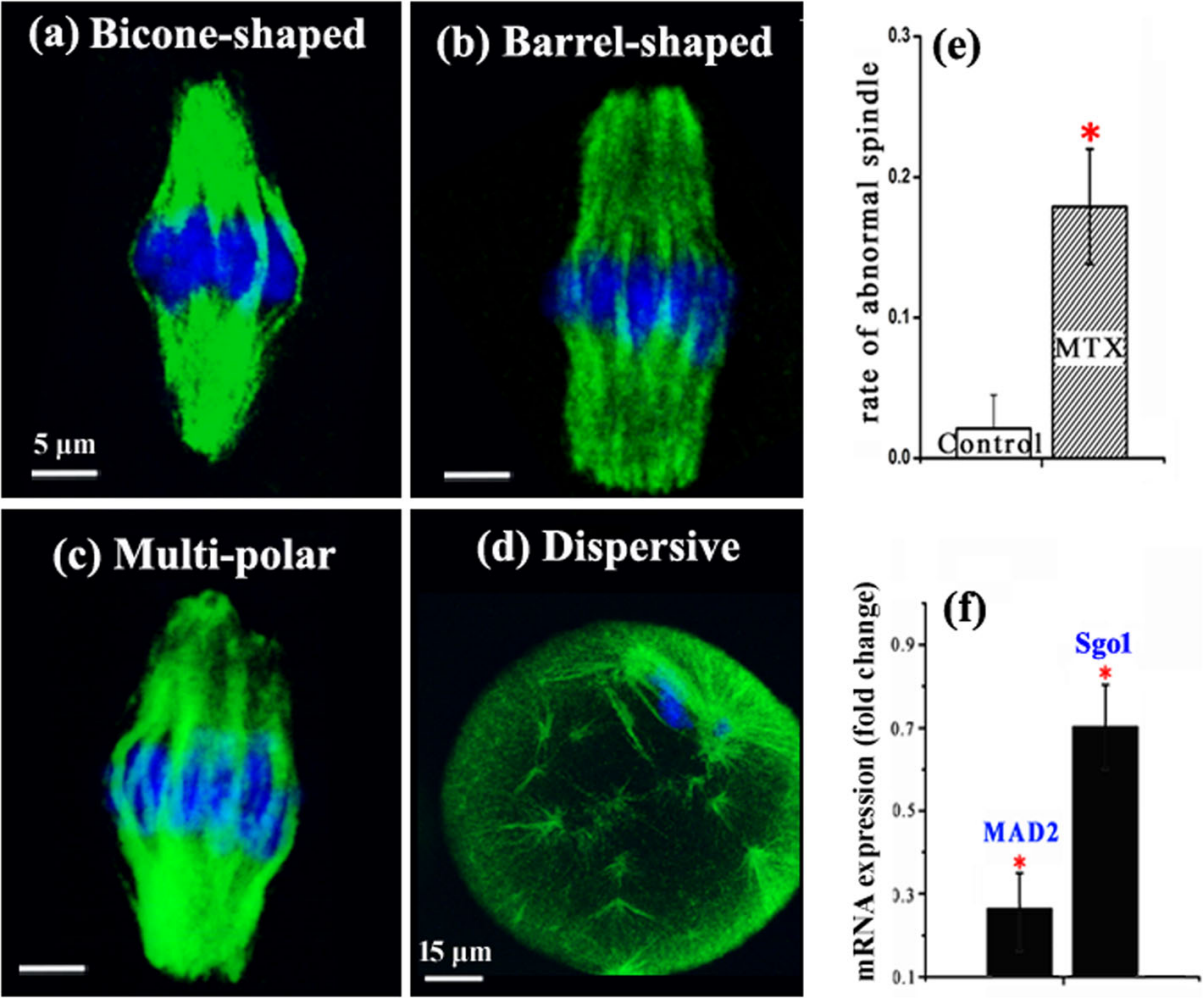
Effects of MTX on the meiotic spindles and transcripts for MAD2 and Sgo1. Oocytes were labelled with antibodies against β-tubulin (green) and were counterstained with Hoechst 33342 (blue). Different spindle morphologies are shown in **a-d**. The rates of abnormal spindle are shown in **e**. mRNA transcript levels of MAD2 and Sgo1 are shown in **f**. The oocyte numbers for analyzing the rates of abnormal spindle were 384 in the control group, and 275 oocytes in the MTX group. * *p*<0.05

The meiotic spindle morphologies and mRNA levels of MAD2 and Sgo1 were examined to analyze the effects of MTX on centromere-associated regulatory facts in chromosome segregation and spindle assembly checkpoint. In this work, we observed three types of spindles (Fig. 3a-d): bipolar spindle (further divided into bicone-shaped spindle, barrel-shaped spindle), multi-polar spindle and dispersive spindle. Multi-polar spindle and dispersive spindle were defined as abnormal morphology. The rate of abnormal spindles in the MTX group was much higher than that in the control group (17.7% ± 4.2% versus 1.9% ± 2.5%; Fig. 3e). Real-time PCR results showed that the transcript levels of MAD2 and Sgo1 were much lower in the MTX group than the control group (0.262 ± 0.093, 0.697 ± 0.129 versus 1.0; Fig.3f).

### MTX altered the DNA methylation modification

Fluorescence polarization was used to determine the global DNA methylation level (that is a simple and new method). It was found that the global DNA methylation level in the MTX group was 32.7% ± 3.4%, much lower than 41.2% ± 2.57% in the control group. Besides, we also assessed DNA methylation level by the traditional method of immunostaining for 5MeC, and found the same results that the relative fluorescence intensity of 5MeC in the MTX group was much lower than that in the control group (0.696 ± 0.139 vs 1.0; Supplementary materials).

**Fig. 4:**
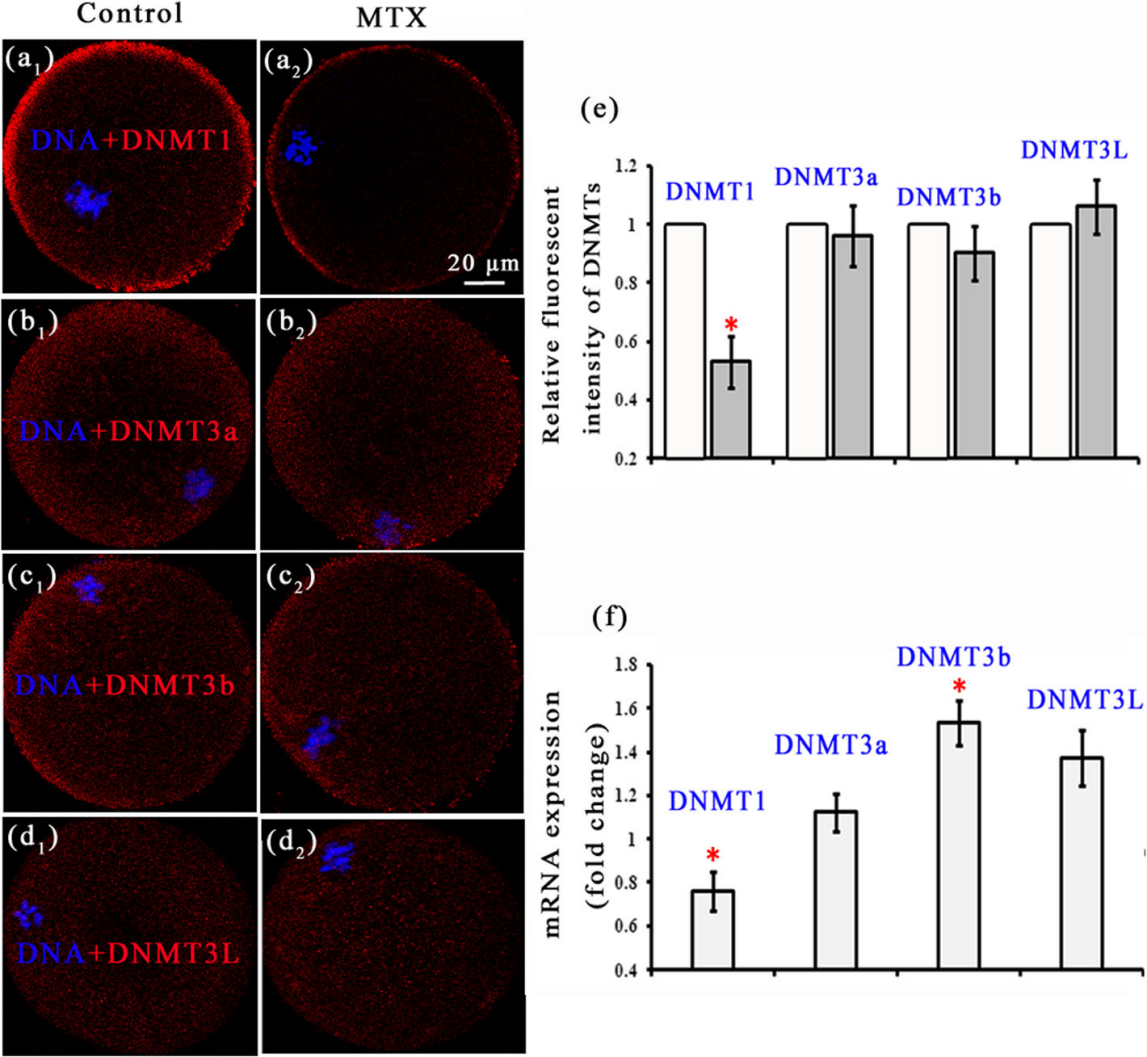
Localizations and transcript expressions of DNMTs. **a-d** show the localizations of DNMT1, DNMT3a, DNMT3b and DNMT3L. Their relative fluorescent intensities are shown in **e**. mRNA transcript levels are shown in **f**. In the control group, 81, 71, 74 and 83 oocytes were used to calculate the relative fluorescent intensities of DNMT1, DNMT3a, DNMT3b and DNMT3L. The corresponding oocytes numbers in the MTX group were 69, 74, 72 and 75. * *p*<0.05

Further, we examined the expressions of DNMTs, and found that in both MTX and control group, DNMT1 in the in-vivo matured oocyte was mainly localized in the cortical region, however, DNMT3a, 3b, 3 L were uniformly distributed in the ooplasm (Fig. 4a-d). The relative fluorescence intensity of DNMT1 in the MTX group was much lower than that in the control group (0.536 ± 0.089 versus 1.0; Fig.4e), while no significant differences were found between the fluorescence intensities of DNMT3a, 3b and 3 L in the MTX and control groups (0.961 ± 0.104, 0.906 ± 0.093, 1.06 ± 0.094 versus 1.0; Fig.4e). Compared with the control group, the mRNA expression for DNMT1 in the MTX group was lower (0.763 ± 0.091 versus 1.0; Fig.4f), while the mRNA expression for DNMT3b was higher (1.536 ± 0.103 versus 1.0; Fig.4f), the others had no obvious differences (1.120 ± 0.086, 1.37 ± 0.127 versus 1.0; Fig.4f).

Since it was not possible to directly analyze the levels of SAM and SAH in the matured oocytes, their concentrations in mouse eyeball blood were measured to obtain some general information on methyl donor availability. The SAM level in the blood was 8.1 ± 1.5 μmol/L in the MTX group, which was not significantly different from 8.2 ± 1.0 μmol/L in the control group. The SAH level in the MTX group was 4.2 ± 0.9 μmol/L, much higher than 1.8 ± 0.7 μmol/L in the control group. The ratios of SAM to SAH were about 1.91 in the MTX group and 4.67 in the control group.

## Discussion

MTX, a specific antifolate drug, affects the folate metabolism via competitively inhibiting DHFR activity [1, 2]. Folate pathway includes 2 cycles which are closely related with DNA and RNA synthesis and methylation modification respectively [1, 5]. Both DNA/RNA synthesis and methylation reaction are crucial for oocyte quality and embryonic development [31–33]. However, does MTX affect the oocyte quality? How does MTX affect the oocyte quality ultimately resulting in the female fertility difficulty (including nature pregnancy and artificial insemination)? Up to now, we still lack the related information. This work focused on this issue.

The rate of abnormal chromosome alignment increased with the dosage of MTX (Table S1). When the dosage of MTX was more than or equal to 5 mg/Kg, the rate of abnormal chromosome alignment reached a plateau and kept steady at about 34%. There were no significant differences in the rate of abnormal chromosome alignment between the groups of MTX dosage more than or equal to 5 mg/Kg. The concentrations of intracellular MTX and its metabolite MTX polyglutamates (MTXPG) are crucial for the efficacy of MTX [34]. Due to the saturation of MTX receptor and MTXPG formation, high-dose MTX may not induce higher intracellular concentrations of MTX and MTXPG. According to our dose-response relationship, the MTX dosage of 5 mg/Kg caused the significant chromosome alignment abnormalities, while the higher MTX dosage (> 5 mg/Kg) didn’t induce more chromosome abnormalities. Thus, this work used the administration of single injection of 5 mg/Kg MTX to establish the MTX model mice for further study. Based on the surface area of animal, the dose of 5 mg/kg for mice equals to 0.4 mg/Kg for human. Referring to medication instructions of MTX, the 0.4 mg/Kg MTX for human is a common dose for treating choriocarcinoma, trophoblastic disease, adult lymphoma, osteosarcoma and other diseases.

This work indicated that MTX impaired the oocyte quality, showing the decreased fertilization rate and a decline in the resulting embryo development potential (Table 1). To explore the possible reasons for MTX-induced damages on oocytes, we firstly examined the influences of MTX on the folate cycles and found that the transcripts for genes encoding key enzymes in the folate cycle were reduced in the MTX group compared with the control group (Fig. 1). This is consistent with the reduced transcript expressions in MTX-treated mouse embryos [14]. The low transcripts suggest that there are disturbances in the one-carbon metabolism and in methyl donor availability [35].

Furthermore, we found that MTX increased abnormalities in chromosome alignment and increased chromosome clustering (Fig. 2). Trencsenyi et al. also found differences in chromatin conformation [36]. The correct separation of chromosomes is crucial for gamete formation and embryo development, the errors during separation will result in serious anomalies, for example, aneuploidy [25]. Chromosome separations are regulated by the spindle formation, the establishment of spindle assembly checkpoint (SAC) and cohesin dissociation [25, 26]. This work found that MTX disrupted the spindle formation showing the increased rate of abnormal spindle morphology (Fig. 3). MAD2, a key protein for SAC, plays a significant role in ensuring chromosome arranging neatly and the coming separation [25, 26]. The loss of MAD2 can cause that chromosomes separate in advance before ranking at equatorial plate [37]. This work found that MTX reduced the mRNA transcript expression of MAD2 (Fig. 3). This indicated that MTX may disturb SAC signalling and proper chromosome separation. Shugoshins are known to protect chromosome cohesion [38]. Reduced Sgo1 at centromeres was associated with precocious separation of chromatids and reduced the embryo development potential [27]. The PCR result in this work showed that the amount of Sgo1 mRNA was significantly reduced in the MTX group (Fig. 3). This indicated that MTX may cause the cohesin dissociation errors and the impediment of chromosome separation via affecting the Sgo1 expression. Thus, we speculated that MTX induced a decline in oocyte fertilization and embryo development potential via disturbing the regulation of chromosome separation.

DNA methylation disorders can result in genomic instability and gene expression disruption [28, 29]. DNA methylation levels in the imprinted domains would change during carcinogenesis [39]. Epigenetic modifications had a close link with the developmental capacity of oocytes and embryos [40]. MTX-induced global DNA methylation reduction suggests that MTX may affect the genome stability and the establishment of imprinted gene in oocytes and reduce the oocyte quality.

DNA methylation is performed by DNMTs which add a methyl to the carbon 5 position of CpG dinucleotide from SAM (methyl donor) [30]. Thus, we examined the expressions and transcript levels of DNMTs. Unlike DNMT3 family scattering in the ooplasmic, DNMT1 mainly localized at the cortical region of ooplasmic (Fig. 4). Compared with the control group, the fluorescence intensity and mRNA transcript level of DNMT1 both were reduced in the MTX group (Fig. 4). Many researches have demonstrated that the down-regulation of DNMT1 could induce the hypomethylation [41–43]. The fluorescence intensities and mRNA transcript levels of DNMT3 family basically had no obvious difference between the MTX and control groups, except the transcript of DNMT3b which greatly increased in the MTX group (Fig. 4). The mRNA level of DNMT3b was found to be inconsistent with its protein expression (Fig. 4). This may be due to the MTX-induced changes on regulation of translation from RNA to protein. Sandhu R’s team showed that altered post-transcription regulation of DNMT 3b by microRNAs affected its expression [44]. Our results of DNMTs might suggest that MTX had little effects on the DNMTs localizations but affected the DNMT1 and DNMT3b transcript levels. It was indicated that DNMT1 and DNMT3b co-regulated the DNA methylation [45, 46]. Specifically, depletion of DNMT1 resulted in widespread hypomethylation and DNMT3b knockdown caused that more hypermethylation than hypomethylation events occurred [45]. In this work, the MTX-induced reduction in the global DNA methylation may be due to the co-regulation of DNMT1 and DNMT3b. Further, we measured the concentrations of SAM and its metabolite SAH in the eyeball blood using HPLC as a rough estimate for the oocytes and found that the ratio of SAM to SAH in the blood was decreased in the MTX group. This ratio was a key indicator to assess the methylation level [47]. It is known that blood provides nutrients to the cells and carries away their wastes. The decreased ratio of SAM to SAH in the blood may signify that this ratio in the oocytes was also reduced. This indicated that MTX changed the methyl donor metabolism. Thus, it was reasonable to assume that the low DNA methylation in the MTX group was also related to the reduced ratio of SAM to SAH.

## Conclusions

This work is the first to confirm the MTX-induced reduction in the in-vivo matured oocyte quality. We showed that MTX impaired the in-vitro fertilization function and the resulting embryo development potential. Further, our observations suggested that MTX disordered the folate metabolism pathway, increased the instabilities of chromosome alignment, and reduced the global DNA methylation level. In short, MTX had side effects on the in-vivo matured oocyte quality which resulted in the poor female fertility.

## Methods

### Experimental design

As shown in Fig. S2, the control and MTX model mice were established by treating with normal saline and 5 mg/kg MTX. (The establishment of the effective dosage of MTX referred to Table S1.) This work studied the in-vivo matured oocytes. After retrieving the in-vivo matured oocytes from the control and MTX model mice, the in-vitro fertilization ability and the resulting embryo formation were firstly analyzed to confirm the effects of MTX on the oocyte quality. Then, the action mechanisms of MTX impairing oocytes quality were studied. From morphological point, fluorescence staining and imaging were used to examine the chromosome morphology, DNMT localization and spindle morphology. From the point of molecular biology, real-time PCR was used to examine the transcripts for genes of the key enzymes in the folate pathway, DNMTs, as well as those in the spindle assembly checkpoint and chromosome segregation, MAD2 and Sgo1. Additionally, we quantitatively measured the global DNA methylation using fluorescence polarization and measured the concentrations of SAM and SAH in the blood using high performance liquid chromatography (HPLC). Through the research above, we analyzed the effects of MTX on the in-vivo matured oocyte quality and its possible mechanisms.

### Animals

The ICR mice (Chinese Academy of Medical Sciences, Beijing, China) were housed and bred in barrier environment. Food and water were available ad libitum according to the Chinese National Standard (GB 14925–2001).

### Oocyte collection

24 h after the treatment with MTX or normal saline, the model mice were treated with 10 IU pregnant mare serum gonadotropin (Bo’ en Pharmaceutical Ltd., Chifeng, China), and after 48 h, were treated with 10 IU human chorionic gonadotropin (Livzon Pharmaceutical Group Inc., Zhuhai, China), then, were euthanized by cervical dislocation 15 h later. Cumulus-oocyte complexes (COCs) were harvested from the oviducts. Cumulus cells were isolated by brief incubation in 0.05% hyaluronidase (Sigma, Louis, MO, USA) at 37 °C for 5 min. This work selected the in-vivo matured oocytes for further study, and these selected oocytes had the features of arresting at the second meiotic metaphase, presenting morphology integrity and good refraction, showing the first polar body*.*

### In-vitro fertilization, embryo culture and morphological examination

ICR male mice were euthanized by cervical dislocation, and their epididymal tails were removed and transferred to HTF droplets. Epididymal tails were punctured to slowly extrude sperm. Then, the sperm suspensions were capacitated in HTF droplets for 90 min. The capacitated sperms were added to the droplets containing COCs and cultured for 6 h. After washing, all the oocytes were transferred to KSOM (Millipore, Massachusetts, USA) droplets and cultured to the blastocyst stage for 4 days. The fertilization and embryo development were recorded by noninvasive imaging technology, full-field optical coherence tomography (FF-OCT), due to the advantages of label-free, three-dimension and high resolution. Fertilization was identified by the appearance of male and female pronuclei. Like our previous work [48], blastomere number was counted by the segmented nuclei which were the dark areas in the images captured by FF-OCT. Additionally, the blastomere localizations were used as a coarse retrieval to distinguish the inner cell mass (ICM) and trophoblast [48].

### Immunofluorescence, two-photon fluorescence imaging and fluorescence intensity analysis

The in-vivo matured nude oocytes were fixed in 4% paraformaldehyde in phosphatebuffered saline (PBS) for 30 min. Then, these fixed oocytes were permeabilized with 0.5% Triton-X 100 for 30 min, and were blocked in 2% normal rabbit serum blocking solution for 30 min. For DNMTs staining, they were incubated with anti-DNMT1, anti-DNMT3a, anti-DNMT3b and anti-DNMT3L (Jackson ImmunoResearch Laboratories, West Grove, USA) overnight at 4 °C in darkness. After washing, the oocytes were immersed in a biotinylated goat anti-rabbit IgG (Jackson ImmunoResearch Laboratories, West Grove, USA) for 30 min. Finally, these oocytes were rinsed and reacted with quantum dot 605-streptavidin conjugate (Invitrogen, California, USA) for 1 h. For spindle labeling, the blocked oocytes were incubated with FITC-conjugated anti-β-tubulin antibody (Sigma, Saint Louis, USA) in darkness for 1 h. Chromosomes were counterstained with Hoechst 33342 (2 μg/mL) for 15 min. The fluorescence images were captured by a Bio-Rad MRC 1024 system (Bio-Rad, California, USA) coupled to a Nikon TE300 inverted microscope (Nikon, Tokyo, Japan) with 100× oil objective (Plan Apochromat DIC H, NA 1.4; Nikon, Tokyo, Japan). All the groups experienced the same experimental conditions (namely, the same excitation laser power and wavelength, detector gain). We used software Amira 5.2 to assess the fluorescence intensity which was defined as the average grey value in the three-dimensional spatial region of interest (DNMTs). According to the method of Zhu [49], the fluorescence intensity in the control group was normalized to one, then, the relative fluorescence intensity in the MTX group was obtained.

### Analysis of mRNA transcript levels

We used RNeasy Micro Kit (Qiagen, California, USA) to extract the total mRNA from about 35 oocytes. Then, RNA purity was estimated by ultraviolet-visible spectrophotometer and its integrity was detected by gel electrophoresis. Total RNA was reverse transcribed using Sensiscript RT Kit (Qiagen, California, USA). According to the manufacturer’s instructions, reverse transcription was performed using 20 μl reactions containing 2 μl 10× buffer RT, 2 μl dNTP Mix, 2 μl Oligo-dT primer, 1 μl RNase inhibitor, 1 μl Sensiscript Reverse Transcriptase, 7 μl RNase-free water, 7 ng RNA template. Then, the 20 μl mixture was placed in a constant temperature bath at 37 °C for 60 min, and the cDNA was obtained. Each analysis was performed for three times using 15 μl reactions containing 7.5 μl SYBR Green PCR Master Mix (Applied Biosystems, UK), 0.6 μl forward and reverse primers (10 μmol/L), 1.5 μl cDNA, and 5.4 μl RNase free water. The sequences of the forward and reverse primers are shown in Table S2. The relative quantitation calculation was implemented by the 2^-△△Ct^ method. β-actin was used as the internal reference.

### Quantitative determination of global DNA methylation

The matured nude oocytes were immersed in Tyrode’s solution (Haling Biotechnology, Shanghai, China) to remove zona pellucid. After washing, these zona-free oocytes were immersed in the lysis buffer (Solarbio, Beijing, China) at 37 °C for 60 min. After sufficient dissociation, the mixed oocyte lysates were centrifuged 10,000 rpm for 2 min, and then saved at − 80 °C.

According to the method of Zhao and Xue [50], a sample of 500 ng DNA was incubated at 37 °C for 12 h in a 50 μl reaction mixture containing 2.5 μl restriction enzyme *H*_*pa*II_ (10 U/μg, TaKaRa Biotechnology, Dalian, China), 5 μl 10× Buffer T, 5 μl 0.1% BSA (Solarbio, Beijing, China), 500 ng DNA, and distilled water adding up to 50 μl. Another sample of 500 ng DNA was incubated at the same conditions, but *H*_*pa*II_ in the reaction mixture was replaced by *M*_*sp*I_ (TaKaRa Biotechnology, Dalian, China). After enzyme digestion, 4 μl enzymatic products of *H*_*pa*II_/*M*_*sp*I_ were placed at 57.8 °C for 60 min in a 15 μl chain-extension reaction mixture containing 1.5 μl 10 × PCR Buffer, 0.63 μl 0.75 U Taq DNA polymerase (TaKaRa Biotechnology, Dalian, China), 0.56 μl TAMRA-dCTP (Applied Biosystems, UK), 4.0 μl restricted DNA and 8.3 μl distilled water. After TAMRA-dCMP incorporating into DNA, the chain-extension reaction products were measured by fluorescence polarization using microplate reader (Molecular SpectraMax Plus). The fluorescence polarization values of *H*_*pa*II_/*M*_*sp*I_ restriction products were recorded as FP_*Hpa*II_ and FP_*Msp*I_. The global DNA methylation level of oocytes was calculated by (1- FP_*Hpa*II_ / FP_*Msp*I_) × 100%.

### Quantitative measurement of SAM/SAH concentration in the eyeball blood

The concentration of SAM and SAH were measurement by extracting the mouse eyeball blood. According to the Zhen’s method [51], 100 μl plasma sample were extracted with 10 μl of 35% perchloric acid. Then, the acidified sample was vortexed for 1 min, subsequently, was placed at about 25 °C for 10 min, lastly, were centrifuged with the speed of 15,000 rpm at 4 °C for 10 min. Ultimately, 25 μl supernatant was analyzed using HPLC. HPLC equipment was Agilent 1100. The chromatogram column (Agilent ZORBAX C8, 250 × 4.6 mm, 5 μm) was used for the analysis. The mobile phase consisted of two solvents: solvent 1 was 8 mM octane sulphonic acid and 50 mM NaH_2_PO_4_ (PH 3.0); solvent 2 was chromatographic grade methanol. The flow rate was 0.9 mL/min. The column temperature was 35 °C. The UV detection wavelength was set at 450 nm from 0 to 8 min, and 254 nm from 8 to 16 min. The SAM/SAH standards configuring in water to different concentrations were used to trace the radio-labelled peaks.

### Statistical analysis

All the experiments were repeated three times. Results were presented as the means ± SEM. Statistical comparisons were performed by One-way ANOVA or chi-square using software SPSS 16.0. *p* < 0.05 was considered significant.

## Supplementary information

**Additional file 1 Figure S1**. Folate metabolism pathway. The key metabolites and enzymes are listed in the folate cycle. Abbreviations (full name): FR (folate receptor), RFC (reduced folate carrier), DHF (dihydrofolate), DHFR (dihydrofolate reductase), THF (tetrahydrofolate), SHMT (serine hydroxymethyl transferase), MTHFR (5,10-methylene THF reductase), MTRR (methionine synthase reductase), MS (methionine synthetase), MAT (methionine adenosyl transferase), SAM (S-adenosylmethionine), DNMT (DNA methyltransferase), SAH (S-adenosylhomocysteine), SAHH (SAH-hydrolase), Hcy (homocysteine), CSB (cystathionine β-synthase).

**Additional file 2 Figure S2**. Experimental technology roadmap.

**Additional file 3 Table S1**. Toxicity assessment of different doses MTX In the control group, mice were treated with normal saline. And the mice in the MTX group were treated with one dose of MTX of 0.5 mg/kg, 5 mg/kg, 10 mg/kg, 20 mg/kg and 50 mg/kg. We analyzed 7 mice in each group. The body weight, the retrieved oocyte number and the rate of chromosome alignment abnormity were used to assess the toxicity of MTX. Specifically, the retrieved oocytes were the in-vivo matured oocytes with the first polar bodies. It was found that there were no obvious differences in the body weight and the retrieved oocytes number between the control group and different doses MTX groups. Compared with the control group, 0.5 mg/Kg MTX had little effects on the chromosome alignment, but the rates of abnormal chromosome alignment in 5 mg/Kg, 10 mg/kg, 20 mg/kg and 50 mg/kg MTX groups were much higher. *p < 0.05. This indicated the administration of single injection of 5 mg/Kg MTX affected oocyte quality showing chromosome instability. Thus, this work used the administration of single injection of 5 mg/Kg MTX to establish the MTX model mice for further study.

**Additional file 4 Table S2**. Primers used for the real time PCR analysis

**Additional file 5:** Supplementary Materials. The details of examining the global DNA methylation via immunostaining for 5MeC.

## Data Availability

The datasets generated and/or analyzed during the current study are available from the corresponding author on reasonable request.

## Abbreviations

MTX: Methotrexate
SLC19A: Solute carrier family 19
DHFR: Dihydrofolate reductase
DHF: Dihydrofolate
THF: Tetrahydrofolate
SHMT: Serinehydroxymethyl transferase
MTHFR: 5,10-methylene THF reductase
MTRR: Methionine synthase reductase
MAT: Methionine adenosyl transferase
CSB: Cystathion β-synthase
SAM: S-adenosylmethionine
SAC: Spindle assembly checkpoint
MAD2: Mitotic arrest deficient 2
Sgo1: Shugoshin 1
DNMTs: DNA methyltransferases
SAH: S-adenosylhomocysteine
HPLC: High performance liquid chromatography
COCs: Cumulus-oocyte complexes
FF-OCT: Full-field optical coherence tomography
ICM: Inner cell mass
PBS: Phosphatebuffered saline
