# Supplementary figures and images for: Methotrexate impaired in-vivo matured mouse oocyte quality and the possible mechanisms
Source: BMC Mol Cell Biol. 2020 Jul 3;21:51. doi: 10.1186/s12860-020-00298-7 (PMC7333412; doi:10.1186/s12860-020-00298-7)

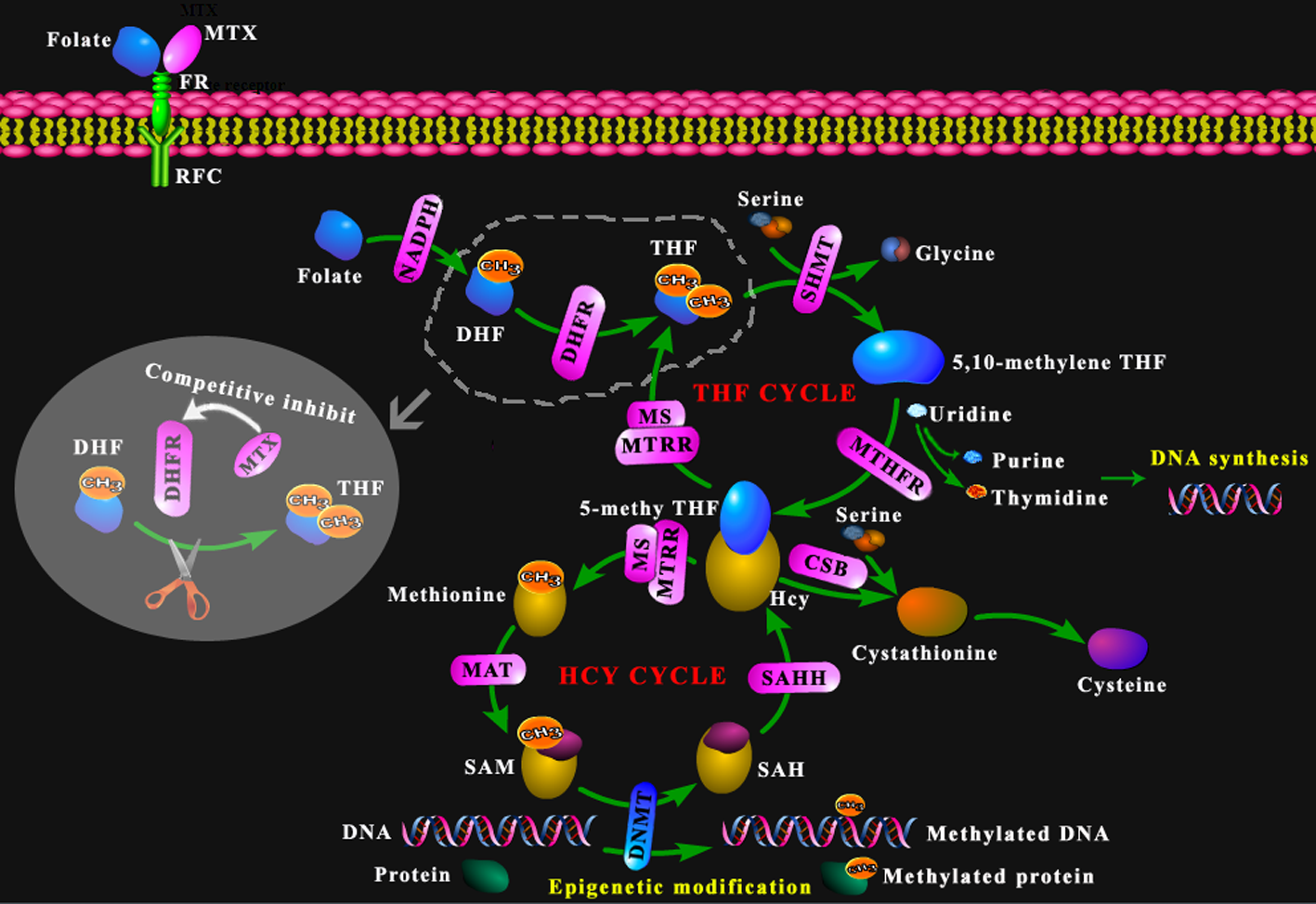

Supplement: Supplementary file 1 — Additional file 1 Figure S1. Folate metabolism pathway. The key metabolites and enzymes are listed in the folate cycle. Abbreviations (full name): FR (folate receptor), RFC (reduced folate carrier), DHF (dihydrofolate), DHFR (dihydrofolate reductase), THF (tetrahydrofolate), SHMT (serine hydroxymethyl transferase), MTHFR (5,10-methylene THF reductase), MTRR (methionine synthase reductase), MS (methionine synthetase), MAT (methionine adenosyl transferase), SAM (S-adenosylmethionine), DNMT (DNA methyltransferase), SAH (S-adenosylhomocysteine), SAHH (SAH-hydrolase), Hcy (homocysteine), CSB (cystathionine β-synthase). [file 12860_2020_298_MOESM1_ESM.tif]

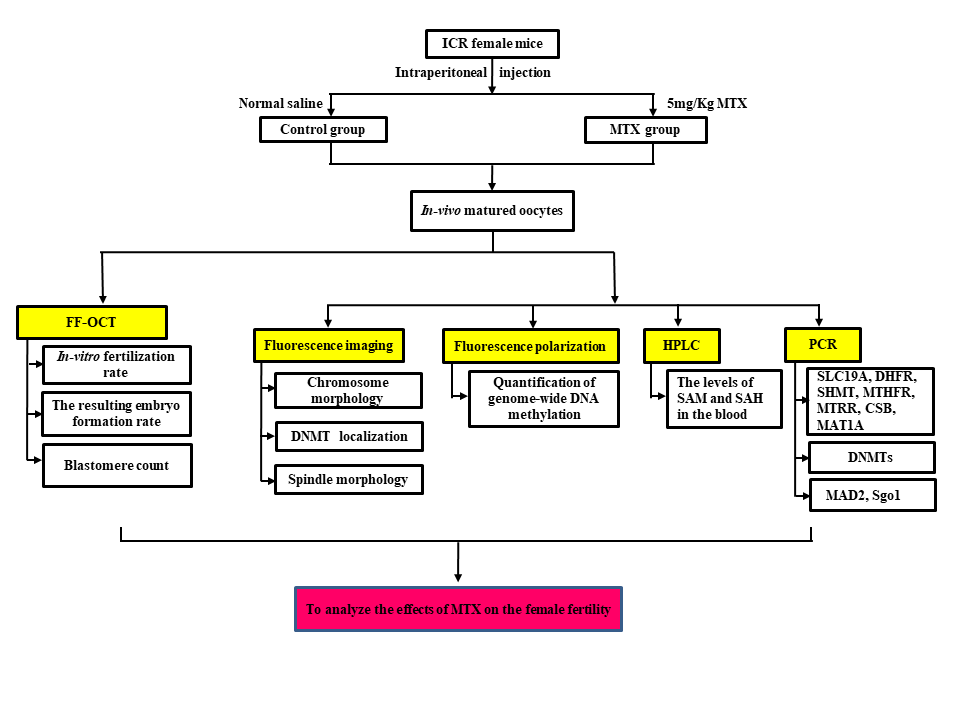

Supplement: Supplementary file 2 — Additional file 2 Figure S2. Experimental technology roadmap. [file 12860_2020_298_MOESM2_ESM.tif]
