# Supplementary material for: Methotrexate impaired in-vivo matured mouse oocyte quality and the possible mechanisms
Source: BMC Mol Cell Biol. 2020 Jul 3;21:51. doi: 10.1186/s12860-020-00298-7 (PMC7333412; doi:10.1186/s12860-020-00298-7)
Supplement: Supplementary file 3 — Additional file 3 Table S1. Toxicity assessment of different doses MTX In the control group, mice were treated with normal saline. And the mice in the MTX group were treated with one dose of MTX of 0.5 mg/kg, 5 mg/kg, 10 mg/kg, 20 mg/kg and 50 mg/kg. We analyzed 7 mice in each group. The body weight, the retrieved oocyte number and the rate of chromosome alignment abnormity were used to assess the toxicity of MTX. Specifically, the retrieved oocytes were the in-vivo matured oocytes with the first polar bodies. It was found that there were no obvious differences in the body weight and the retrieved oocytes number between the control group and different doses MTX groups. Compared with the control group, 0.5 mg/Kg MTX had little effects on the chromosome alignment, but the rates of abnormal chromosome alignment in 5 mg/Kg, 10 mg/kg, 20 mg/kg and 50 mg/kg MTX groups were much higher. *p < 0.05. This indicated the administration of single injection of 5 mg/Kg MTX affected oocyte quality showing chromosome instability. Thus, this work used the administration of single injection of 5 mg/Kg MTX to establish the MTX model mice for further study. [file 12860_2020_298_MOESM3_ESM.doc]

Table S1. Toxicity assessment of different doses MTX

|  | Control group | MTX group | | | | | | | |
| --- | --- | --- | --- | --- | --- | --- | --- | --- | --- |
|  |  | 0.5 mg/kg | 5 mg/kg | | 10 mg/kg | | 20 mg/kg | | 50 mg/kg |
| Number of mice for experiment | 7 | 7 | | 7 | | 7 | | 7 | 7 |
| Average body weight of mice (g) | 22.6±3.2 | 23.1±3.4 | | 21.9±2.5 | | 22.4±2.1 | | 21.7±3.6 | 21.3±2.8 |
| Average number of retrieved oocytes | 10.4±3.6 | 10.4±4.5 | | 9.7±4.3 | | 10.3±3.8 | | 10.4±4.1 | 10.2±4.3 |
| Rate of abnormal chromosome alignment | 12.6% | 15.4% | | 33.2%* | | 34.3%* | | 34.4%* | 33.1%* |

In the control group, mice were treated with normal saline. And the mice in the MTX group were treated with one dose of MTX of 0.5 mg/kg, 5 mg/kg, 10 mg/kg, 20 mg/kg and 50 mg/kg. We analyzed 7 mice in each group. The body weight, the retrieved oocyte number and the rate of chromosome alignment abnormity were used to assess the toxicity of MTX. Specifically, the retrieved oocytes were the *in-vivo* matured oocytes with the first polar bodies. It was found that there were no obvious differences in the body weight and the retrieved oocytes number between the control group and different doses MTX groups. Compared with the control group, 0.5 mg/Kg MTX had little effects on the chromosome alignment, but the rates of abnormal chromosome alignment in 5 mg/Kg, 10 mg/kg, 20 mg/kg and 50 mg/kg MTX groups were much higher. *p < 0.05. This indicated the administration of single injection of 5 mg/Kg MTX affected oocyte quality showing chromosome instability. Thus, this work used the administration of single injection of 5 mg/Kg MTX to establish the MTX model mice for further study.
