# Supplementary material for: Methotrexate impaired in-vivo matured mouse oocyte quality and the possible mechanisms
Source: BMC Mol Cell Biol. 2020 Jul 3;21:51. doi: 10.1186/s12860-020-00298-7 (PMC7333412; doi:10.1186/s12860-020-00298-7)
Supplement: Supplementary file 4 — Additional file 4 Table S2. Primers used for the real time PCR analysis [file 12860_2020_298_MOESM4_ESM.doc]

Table S2. Primers used for the real time PCR analysis

| Gene | Forward primer sequence | Reverse primer sequence |
| --- | --- | --- |
| SLC19A | 5’-gcgatgcctggtgttctatct-3’ | 5’-cggaatgatctcgttagtcacct-3’ |
| DHFR | 5’-agaacggagacctaccctgg-3’ | 5’-gaggttgtggtcattctttggaa-3’ |
| SHMT1 | 5’-gggtcggattagagctgattg-3’ | 5’-ggtcccgccataatacctttg-3’ |
| SHMT2 | 5’-tgactatgcacgcatgagaga-3’ | 5’-atccgcgtacttgaaagggg-3’ |
| MTHFR | 5’-agatgaggcgcagaatggac-3’ | 5’-catccggtcaaacctggagat-3’ |
| MTRR | 5’-catgccgatgactgtgtcg-3’ | 5’-ccgagtgacttaaagtgctttgt-3’ |
| MAT1A | 5’-ctgtgtggggagatcacctca-3’ | 5’-cccttggcagagtcgtcatag-3’ |
| CSB | 5’-caaagaatgccggtctcaagt-3’ | 5’-ctcagcatcttcgatcatccg-3’ |
| DNMT1 | 5’-cctagttccgtggctacgaggagaa-3’ | 5’-tctctctcctctgcagccgactca-3’ |
| DNMT3a | 5’-acttggagaagcggagtgaa-3’ | 5’-ctgttctttgccctctcctg-3’ |
| DNMT3b | 5’-ttcagtgaccagtcctcagacacgaa-3’ | 5’-tcagaaggctggagacctccctctt-3’ |
| DNMT3L | 5’-gtgcgggtactgagcctttttaga-3’ | 5’-cgacatttgtgacatcttccacgta-3’ |
| MAD2 | 5’-gtggccgagtttttctcatttg-3’ | 5’-aggtgagtccatatttctgcact-3’ |
| Sgo1 | 5’-tggaggtattggttcctgtgatg -3’ | 5’-ctgcattcgaggtcactcacttc -3’ |
