# Supplementary material for: Methotrexate impaired in-vivo matured mouse oocyte quality and the possible mechanisms
Source: BMC Mol Cell Biol. 2020 Jul 3;21:51. doi: 10.1186/s12860-020-00298-7 (PMC7333412; doi:10.1186/s12860-020-00298-7)
Supplement: Supplementary file 5 — Additional file 5: Supplementary Materials. The details of examining the global DNA methylation via immunostaining for 5MeC. [file 12860_2020_298_MOESM5_ESM.pdf]

Supplementary materials

Immunofluorescence

The MII-arrested nude oocytes were fixed in 4% paraformaldehyde in phosphatebuffered saline (PBS) for 30 min. Then, these fixed oocytes were permeabilized with 0.5% Triton-X 100 for 30 min, and, were blocked in 2% normal goat or rabbit serum blocking solution for 30 min. Next, these oocytes were denatured with 4 N HCl for 10 min, and were neutralized with 100 mM Tris-HCl (pH 8.5) for 10 min, then were incubated with anti-5-methyl cytosine antibody (1:100; Abcam, Cambridge, UK). After washing, the oocytes were immersed in a biotinylated goat anti-rabbit or rabbit anti-goat IgG (diluted 1:100 in PBS; Jackson ImmunoResearch Laboratories, West Grove, USA) for 30 min. Finally, these oocytes were rinsed and reacted with quantum dot 605-streptavidin conjugate (diluted 1:50 in PBS; Invitrogen, California, USA) for 1 h. Chromosomes were counterstained with Hoechst 33342 (2 μg/mL) for 15 min.

Imaging

The fluorescence images were captured by a Bio-Rad MRC 1024 system (Bio-Rad, California, USA) coupled to a Nikon TE300 inverted microscope (Nikon, Tokyo, Japan) with 100× oil objective (Plan Apochromat DIC H, NA 1.4; Nikon, Tokyo, Japan).

Fluorescence intensity analysis

All the groups experienced the same experimental conditions (namely, the same excitation laser power and wavelength, detector gain, etc). We used software Amira 5.2 to assess the fluorescence intensity which was defined as the average grey value in the

three-dimensional spatial region of interest (namely, chromosome).

Results

We assessed DNA methylation level by immunostaining for 5MeC. 5MeC level was estimated by the relative fluorescence intensity. In the MTX group, 5MeC level was much lower than that in the control group (0.696 ± 0.139 vs 1.0; $p < 0.05$, Fig. S1).

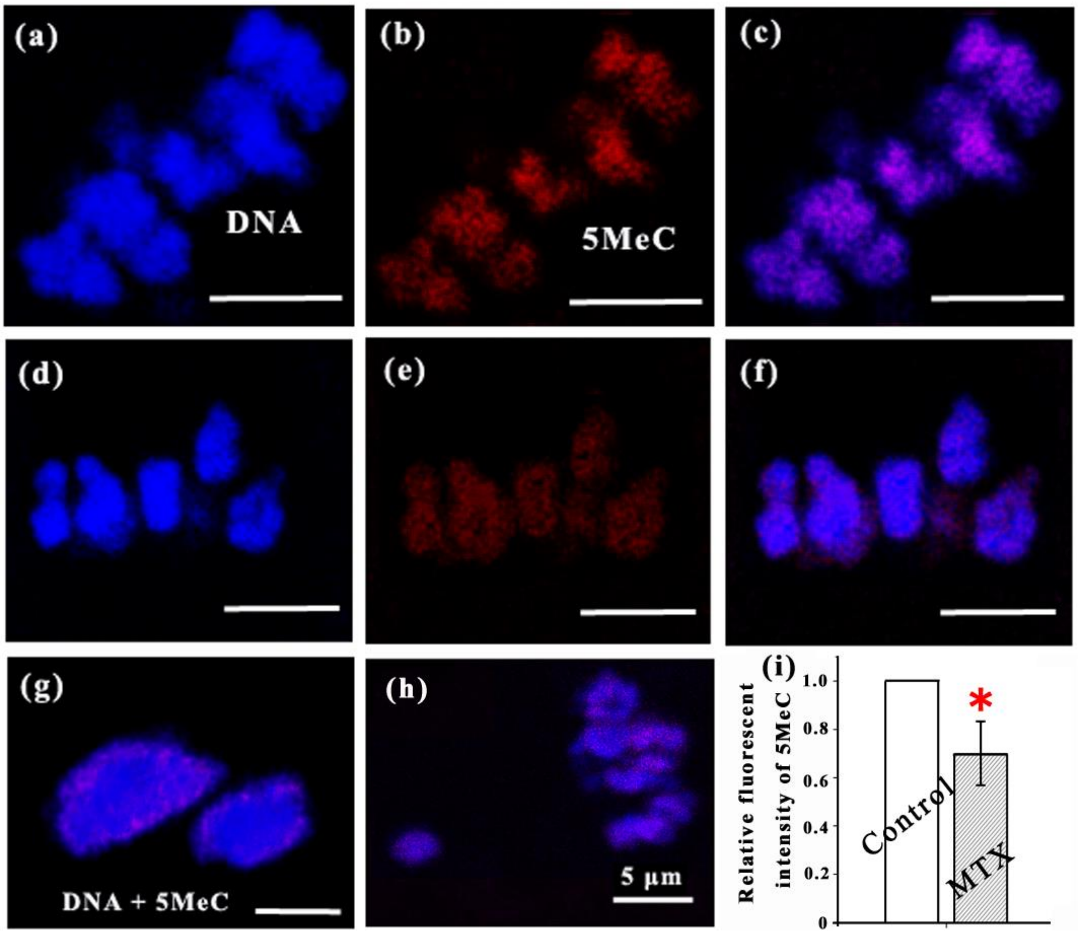

Figure S1. Effects of MTX on DNA methylation. DNA was labeled by Hoechst 33342. DNA methylation was visualized by immunostaining for 5MeC. The images of DNA and DNA methylation in the control group are shown in (a-c), and these images in the MTX group are shown in (d-f). (g, h) also show DNA methylation in the MTX group. The relative fluorescent intensity of 5MeC is shown in (i). n=113 in the control group, and n=92 in the MTX group. *Significantly different ($p < 0.05$).
